# Alarmin Cytokines as Central Regulators of Cutaneous Immunity

**DOI:** 10.3389/fimmu.2022.876515

**Published:** 2022-03-30

**Authors:** Tatsuya Hasegawa, Tomonori Oka, Shadmehr Demehri

**Affiliations:** ^1^ Shiseido Global Innovation Center, Yokohama, Japan; ^2^ Center for Cancer Immunology and Cutaneous Biology Research Center, Department of Dermatology and Center for Cancer Research, Massachusetts General Hospital and Harvard Medical School, Boston, MA, United States

**Keywords:** TSLP, IL-33, IL-25, skin immunlogy, allergic inflammation, cancer, host defense, skin homeostasis

## Abstract

Skin acts as the primary interface between the body and the environment. The skin immune system is composed of a complex network of immune cells and factors that provide the first line of defense against microbial pathogens and environmental insults. Alarmin cytokines mediate an intricate intercellular communication between keratinocytes and immune cells to regulate cutaneous immune responses. Proper functions of the type 2 alarmin cytokines, thymic stromal lymphopoietin (TSLP), interleukin (IL)-25, and IL-33, are paramount to the maintenance of skin homeostasis, and their dysregulation is commonly associated with allergic inflammation. In this review, we discuss recent findings on the complex regulatory network of type 2 alarmin cytokines that control skin immunity and highlight the mechanisms by which these cytokines regulate skin immune responses in host defense, chronic inflammation, and cancer.

## Introduction

Skin is the largest organ and acts as a protective barrier separating the body from the outside environment (1). Epidermis, dermis, and subcutaneous fat together with skin appendages, sweat glands, sebaceous glands, and hair follicles, form an integrated structure that enables proper skin function (2). The foremost physical barrier of the skin consists of the epidermis, which in its outermost layer is composed of brick and mortar-like stratum corneum and tight junctions that regulate the inward and outward passage of fluid and electrolytes in and out of the skin (1, 3). The skin has developed complex protective functions against constant exposure to various environmental insults, such as solar radiation, pollutants, a broad range of microbial pathogens, and allergens (4, 5). The immune system contributes to this first line of defense against microbial pathogens and chemical insults (2).

In the epidermis, Langerhans cells (LCs) are epidermal-resident antigen-presenting cells (APCs), which play a sentinel role as the first professional immune cells confronting the environmental insults (6, 7). Activated LCs capture foreign antigens by extending their dendrites through epidermal tight junctions. Next, they migrate to the lymph nodes to initiate cutaneous adaptive immunity (8, 9). In addition, tissue-resident memory T cells (T_RM_ cells), which are noncirculating lymphocytes in peripheral tissues, persist in the epidermis to provide long-lasting protective defense against future immunological challenges at the most probable sites of invasion (10–12). The dermis is the stromal layer below the epidermis that encompasses an active immunological microenvironment (2). The superficial papillary dermis is composed of a relatively loose connective tissue and contains vessels and sensory nerves (13). A diverse range of immune cell types, including several T cell subsets, macrophages, dendritic cells (DCs), innate lymphoid cells (ILCs), and mast cells, is localized around vasculature and extracellular matrix proteins in the dermis (14–16). This complex network of dermal immune cells helps in many aspects of host physiology, including protection against pathogens, wound healing (17), sebum production (18), and hair follicle homeostasis (19).

Cytokine-mediated communication allows immune cells to achieve a context-appropriate response, as the homeostasis of a multi-cellular organism is made possible by the proper cell-cell communication across different cell types (20). The cytokine superfamily consists of many ligands and receptors that mediate key interactions between immune cells and non-immune cells, including keratinocytes, fibroblasts, and endothelial cells, in the skin microenvironment (21–23). As the primary barrier, which is constantly exposed to environmental insults, the epidermis functions as a key sensor and integrator of environmental cues to regulate immunity in the skin (24, 25). As such, epidermis-derived alarmin cytokines mediate an intricate intercellular communication between epidermal keratinocytes and immune cells to regulate cutaneous immune surveillance. Alarmins are endogenous molecules that function as danger signals and are rapidly released to the extracellular milieu in response to tissue damage to trigger defensive immune responses (26). Among them, the proper functions of type 2 alarmin cytokines, thymic stromal lymphopoietin (TSLP), interleukin (IL)-25 and IL-33, as central orchestrators of T helper 2 (Th2) immunity, are paramount to the skin homeostasis and their dysregulation is commonly associated with chronic allergic inflammation (27–29).

In this review, we discuss recent findings on the complex functions of type 2 alarmin cytokines in regulating epithelium-immune cell communication that governs host defense, chronic inflammation, and cancer.

## TSLP

TSLP is a member of the IL-2 family of cytokines, and its receptor is a heterodimer that consists of the IL-7 receptor α chain (IL-7Rα), which is shared with IL-7, and the TSLP receptor (TSLPR) (30–32). TSLP is mainly expressed by the epithelial cells of the gut, lung, and skin. Other cell types including DCs, basophils, and mast cells can express TSLP (33, 34). At the organ level, TSLP is widely distributed in several organs including the heart, liver, testis, spleen, prostate, skin, lung, kidney, ovary, small intestine, and colon (35). A variety of endogenous and environmental factors, such as pro-inflammatory cytokines, tryptase, invading pathogens, allergens, irritants, pollutants, and cigarette smoke, stimulate epithelial cells to release TSLP at barrier surfaces (36–45). Activation of protease-activated receptor 2 (PAR2), Toll-like receptor 4 (TLR4), and a member of transient receptor potential vanilloid (TRPV) channel family, including TRPV1, on the cell membrane mediate the production of TSLP through transcription factors, nuclear factor of activated T cells (NFAT), nuclear factor-kappa B (NF-κB), and interferon regulatory factor 3 (IRF-3) (46–48). TSLP is post-translationally modified by endogenous proteases, and cleaved TSLP has an increased biological activity (49). Despite poor sequence homology of TSLP (43% amino acid identity) and TSLPR (39% amino acid identity) between humans and mice, TSLP has been shown to exhibit similar biological functions in humans and mice (50).

Hematopoietic cell populations and sensory neurons express TSLPR. TSLP first interacts with the cognate TSLPR, then IL-7Rα can be recruited to the TSLP/TSLPR assembly to form the extracellular ternary complex. This leads to the activation of an intricate network of signaling pathways, including Janus kinase/signal transducer activator of transcription (JAK/STAT) and phosphatidylinositol-3 kinase (PI3K) pathways (51). Similar to IL-7, this signaling plays a critical role in the activation and differentiation of immune cells, such as B cells and T cells (52). In contrast, TSLP also mediates Th2 immunity associated with protection from helminth parasites and the pathogenesis of allergic inflammation at barrier surfaces. TSLP strongly induces the expression of major histocompatibility complex (MHC) class I and II molecules and costimulatory molecules on DCs. TSLP-activated DCs produce Th2-attracting chemokines, such as CCL17 and CCL22 (53), and induce Th2 differentiation through OX40 ligand upregulation on these cells (54, 55). Furthermore, TSLP can directly activate naïve CD4^+^ T cells to differentiate and promote Th2 effector function in a TCR-dependent manner (56–58), indicating that TSLP is a key driver of Th2 immunity. In addition, TSLP acts on CD4^+^ T cells to promote T helper 9 (Th9) differentiation and function through STAT5 activation in airway inflammation (59). Th9 cells, IL-9 producing CD4^+^ T cells, are closely related to Th2 cells and contribute to allergic inflammation and anti-tumor immunity (60, 61). It remains unclear whether these cells represent a truly unique Th cell subset. TSLP also acts on basophils and group 2 ILCs (ILC2s) to induce Th2 responses (62, 63). Thus, TSLP orchestrates type 2 immune responses by innate and adaptive immune cells at barrier sites.

### TSLP in Skin

Keratinocytes are a powerful source of TSLP in the skin under chronic and severe barrier disruption (64, 65). TSLP is released from keratinocytes in response to cutaneous pathogens, such as *Staphylococcus aureus* (66) and *Malassezia* yeasts (67), and environmental stimuli, such as ultraviolet radiation (68), mechanical injury (41), and air pollutants (69). The thermosensitive transient receptor potential channels TRPV3 and TRPV4 (70, 71), pattern recognition receptor TLR3 (72, 73), and PAR2 (38, 74) are among the receptors that can sense the external stimuli and induce the production of TSLP in keratinocytes. Vitamin D3 also induces the expression of TSLP in keratinocytes, which leads to the development of an atopic dermatitis-like phenotype (75). In contrast, TSLP is negatively regulated by retinoid X receptor αβ (RXRαβ) (76), aryl hydrocarbon receptor (AhR) (77), and Notch signaling (64), which function as a gatekeeper in keratinocytes. Chronic TSLP release by keratinocytes responding to cellular and tissue damage instigates type 2 immune response that leads to atopic dermatitis. High TSLP expression is observed in a broad spectrum of skin lesions of atopic dermatitis (53), psoriasis (78), Netherton syndrome (79), and keloid (80). Continuous skin barrier disruption, characterized by atopic dermatitis, facilitates epicutaneous sensitization, which accelerates TSLP expression in keratinocytes. Furthermore, *TSLP* promoter demethylation is detected in skin lesions from patients with atopic dermatitis (81).

TSLPR is broadly expressed by immune cells and sensory neurons in the skin. In particular, DCs are important TSLP-responsive immune cell populations (27, 53–55, 82). Several DC subsets, including epidermal LCs and dermal type 1 and 2 conventional DCs, respond to keratinocytes-derived TSLP signals to initiate cutaneous adaptive immunity and provide multiple soluble and surface-bound signals that help to guide T cell differentiation, in particular Th2 cells (83–86). In atopic dermatitis lesions, TSLP may contribute to the activation of LCs, which then migrate to the draining lymph nodes and prime allergen-specific Th2 responses (53). In addition, TSLP-mediated LC activation can promote the differentiation of CD4^+^ T cells into Th2 and follicular helper T cells (Tfh cells), which are important regulators of humoral responses (87). Moreover, TSLP and transforming growth factor-β1 (TGF-β1) synergistically contribute to the pool of LCs during inflammation *via* the promotion of LC differentiation from human blood BDCA-1^+^ DCs (88). Dermal DCs act as critical responders in TSLP-mediated type 2 allergic inflammatory responses. TSLP activates CCL17-producing CD11b^+^ dermal DCs to migrate to draining lymph nodes and attract naïve CD4^+^ T cells to differentiate into Th2 cells during contact hypersensitivity in mice (89). Furthermore, TSLP-stimulated DCs act not only on the priming of Th2 cells but also on the maintenance and further polarization of Th2 central memory cells in allergic inflammation (90). Besides Th2 priming, TSLP-mediated DC activation conducts multiple CD4^+^ T cell fate specifications in the skin, depending on the surrounding inflammatory microenvironment. TSLP drives the differentiation of IL-21-producing human Tfh cells through OX40 ligand in CD1c^+^ DCs and helps memory B cells to produce immunoglobulin G (IgG) and IgE in a Th2 cell-dominated environment (91). Furthermore, TSLP is highly produced by keratinocytes in patients with psoriasis, where it synergizes with CD40 ligand in skin DCs to promote the expression of the Th17-polarizing cytokine IL-23 (78).

TSLP can directly activate CD4^+^ T cells and induce the differentiation of a distinct population of effector Th2 cells in lymph nodes (58). TSLP/TSLPR signaling amplifies IL-4 production from CD4^+^ T cells, which results in driving a positive feedback loop between TSLP and IL-4 to exacerbate Th2 cell-mediated allergic inflammation (92). In fact, atopic dermatitis patients possess circulating CD4^+^ T cells expressing high TSLPR levels, and the frequency of this subset correlates with the severity of atopic dermatitis (93). In addition, Th2 cells express more TSLPR than Th1 or Th17 cells (94). ILC2s are also important targets of TSLP in allergic inflammation (95). TSLP-elicited ILC2 promotes allergic inflammation, whereas IL-25 and IL-33 are dispensable for this ILC2 response in an atopic dermatitis-like mouse model (95). Although IL-25 and IL-33 are critical mediators to elicit ILC2s in the gut and lung for anti-helminth immunity and allergic inflammation (96), skin-specific ILCs may have distinct properties. Accordingly, transcriptomic heterogeneity of ILC2s is apparent across tissues, and skin-resident ILC2s express relatively low levels of receptors of type 2 cytokines, such as TSLP, IL-25, and IL-33, and instead dominantly express IL-18 receptor (IL-18R1), compared with other tissues (97, 98). Indeed, IL-18 can activate skin ILC2s and synergize with type 2 cytokines in the development of atopic dermatitis-like disease (98). Furthermore, two distinct populations of ILC2s, consisting of skin-resident and circulating ILC2s, exist in the murine skin, and they exhibit distinctive phenotypes and functions (99). The response of ILC2s may ultimately depend on the nature of the inflammatory stimulus in the microenvironment.

Keratinocytes-released TSLP signals directly reach out to sensory neurons in the skin. PAR2-triggered release of TSLP can stimulate sensory neurons to evoke the itch response in allergic diseases such as atopic dermatitis, in a TSLPR- and TRPA1-dependent manner (38). In addition, TRPV4 triggers TSLP release, which activates sensory neurons through TSLPR and TRPV4 in a dry skin-induced pruritus model (71). TSLP is found to be involved in the later phase of itch progression in allergic inflammation while neutrophil-derived CXCL10 drives itch in the acute phase, which is mediated through CXCR3 on sensory neurons (100). TSLP widely impacts a broad array of dermal immune cells in the skin. TSLP elicits skin basophils, and TSLP-dependent basophil-derived IL-4 promotes ILC2 responses during atopic dermatitis-like inflammation (101). Furthermore, TSLP induces mast cell development through the activation of mouse double minute 2 (MDM2) and STAT6, which results in skin allergic inflammation (45, 102).

Excessive TSLP that is secreted by barrier-defective skin into the systemic circulation leads to sensitization of the lung airways to inhaled allergens characterized by allergic asthma-like phenotype in mice (103, 104). Thus, high systemic levels of skin-derived TSLP instigate the atopic march whereas IL-25 does not (105). Meanwhile, regulatory T cell (Treg)-mediated immunosuppression directly by TSLP from keratinocytes protects against progression from a local skin inflammatory response to a lethal systemic condition (106). TSLP has a dual function as a pro-inflammatory and pro-homeostatic modulator, and this may depend at least in part on the nature of surrounding immune signals and the type of cells responding to TSLP in the tissue microenvironment.

## IL-25

IL-25 (also known as IL-17E) belongs to the IL-17 cytokine family, which consists of six members, and shares relatively low sequence similarity to the prototype member, IL-17 (alternative name IL-17A) (107–109). IL-25 receptor (IL-25R) is a heterodimer of the IL-17RA chain, which is shared with other IL-17 family members, and the IL-25-specific IL-17RB chain (108). Therefore, IL-25 exhibits a distinct function from other members of the IL-17 cytokine family and has been implicated as a type 2 cytokine that induces the production of IL-4, IL-5, and IL-13, which in turn inhibit the IL-17-dependent autoimmune diseases (110). Furthermore, IL-25 enhances Th9 cell response to prevent parasitic helminths infection (111, 112). IL-25 is produced by epithelial and immune cells including Th2 cells, macrophages, ILC2s, mast cells, basophils, and eosinophils (109, 113). In the extracellular space, IL-25 has been reported to be a substrate for proteolytic cleavage by matrix metalloproteinase-7 (MMP-7) from airway epithelial cells during inflammation, and cleaved IL-25 increases its activity to induce type 2 cytokines (114). Expression of IL-25R has been reported on non-immune cells, including fibroblasts and endothelial cells, and immune cells, such as Th2 cells, natural killer T cells (NKT cells), DCs, macrophages, ILC2s, mast cells, basophils, and eosinophils in the inflammatory state (115, 116).

### IL-25 in Skin

IL-25 has been reported to be highly expressed in several skin inflammatory diseases, including atopic dermatitis (117), psoriasis (118), pyoderma gangrenosum (119), acute generalized exanthematous pustulosis (119), and cutaneous T-cell lymphoma (CTCL) (120). IL-4, IL-13, IL-22, endothelin-1, and periostin enhance the production of IL-25 from keratinocytes (120–122). IL-25 induces allergic skin inflammation, characterized by elevated expression of IL-4 and IL-5, dermal infiltration of immune cells, epidermal hyperplasia, and impairment of skin barrier function in mice (118). Epidermal keratinocytes-derived IL-25 is a central regulator of a broad array of allergic inflammatory responses, and the major targets of IL-25 are dermal ILC2s and macrophages in the skin (123). IL-25 activates ILC2s to promote IL-13 production, which in turn helps keratinocytes proliferate and produce immune cell-attracting chemokines (124, 125). IL-25 also promotes the recruitment of neutrophils *via* activation of macrophages in a p38-dependent manner (119). On the other hand, IL-25 responds to tissue injury and participates in cutaneous would healing through an amelioration of angiogenesis and collagen deposition in diabetic mice model (126).

The autocrine function of IL-25 in keratinocytes promotes proliferation and inflammatory responses *via* STAT3 transcriptional factor, which results in amplification of psoriatic skin inflammation (118). Unlike IL-17, IL-25 is not capable of inducing antimicrobial peptides, β-defensin 2 and LL-37, in keratinocytes (121). IL-25 acts synergistically with Th2 cytokines, IL-4 and IL-13, to down-regulate filaggrin expression in keratinocytes exacerbating skin barrier defects (127). Down-regulation of filaggrin expression by IL-25 is mediated at least in part through the activation of NF-κB (107), whereas Th2 cytokines activate STAT6 (128, 129). In the fluorescein isothiocyanate-induced contact hypersensitivity model, IL-25 induces hapten-specific Th17 immunity, rather than Th2 immunity, in the elicitation phase of contact hypersensitivity (130). Following the hapten challenge, dermal DCs release IL-1β in response to IL-25, and IL-1β directly activates Th17 cells. This contrasts with the observed role of TSLP in DC activation and hapten-specific Th2 cell differentiation in the sensitization phase of contact hypersensitivity (131). These findings indicate that type 2 alarmin cytokines have distinct mechanisms for the regulation of T cell responses during inflammation.

## IL-33

IL-33 is the most recently discovered member of type 2 alarmin cytokines (132). IL-33 was first described in 2005. It belongs to the IL-1 family of cytokines, which includes IL-1α, IL-1β, IL-18, IL-36α, IL-36β, IL-36γ, and IL-37, and the receptor antagonists IL-1Ra, IL-36Ra, and IL-38 (133). In contrast to its other family members, IL-1 and IL-18, IL-33 has been shown to promote Th2 cytokine responses in helminth infection and allergic inflammation (132). IL-33 is mainly expressed by non-immune cells, including epithelial cells, endothelial cells, and fibroblasts. It can also be expressed by immune cells, including macrophages and mast cells, at the barrier sites, where it functions as an alarmin following tissue damage (134, 135). IL-33 is localized in the cell nucleus, and its N-terminal domain, which includes a chromatin-binding motif, is required for its nuclear localization (136). Unlike IL-1β and IL-18, the N-terminal portion of IL-33 does not require inflammasome-mediated cleavage by caspase-1 for extracellular release of the active form. Apoptosis-associated caspase-3 and caspase-7 cleave and inactivate IL-33 at a conserved residue, Asp^178^ (Asp^175^ in mouse), within the IL-1-like cytokine domain (137). On the other hand, N-terminal processing of extracellular full-length IL-33 can occur in the central domain between the nuclear domain and the IL-1-like cytokine domain. These residues are targeted by extracellular proteases in the inflammatory microenvironment, including neutrophil cathepsin G, neutrophil elastase, and mast cell serine proteases, and the resulting 18-21 kDa cytokine forms of IL-33 exhibit higher biological activity (138, 139). Full-length IL-33 can be rapidly cleaved in its central sensor domain by extracellular environmental allergens-derived proteases within 10-20 minutes (140). In contrast, cysteine oxidation of extracellular IL-33 diminishes its biological activity (141). Thus, following IL-33 release, the impact of IL-33 is tightly regulated by post-translational modifications in the extracellular milieu. Although IL-33 lacks a secretion sequence and is sequestered in the nucleus *via* chromatin binding, IL-33 seems to be released into extracellular space through an unconventional secretion pathway following various stimuli. Environmental allergens, including fungi and mites, and mechanical stress trigger the release of IL-33 (142–145). Two primary scenarios for IL-33 release have been proposed: passive release as alarmin from necrotic cells during tissue damage and unconventional secretion from living cells. A recent study suggests that IL-33 is secreted through the extracellular vesicles pathway, commonly referred to as exosomes, as surface-bound cargo, from living airway epithelial cells (146). However, the molecular mechanisms and pathways of IL-33 secretion in living cells remain unclear.

IL-33 binds to its receptor, suppressor of tumorigenesis 2 (ST2), on target cells (147, 148). ST2 is classified as a member of the IL-1 receptor superfamily, which has a common intracellular domain, known as the Toll/Interleukin-1 receptor (TIR) domain (147, 148). IL-33 signals *via* its cognate receptor ST2, which is highly expressed on Th2 cells, ILC2s, and mast cells, and induces Th2-skewed immunity to help with the removal of invading pathogens and helminths (29, 149). However, the detrimental effects of its chronic expression in response to environmental insults cause allergic inflammation. In addition, inappropriate activation of the IL-33/ST2 axis after tissue injury can lead to impaired wound healing and tissue remodeling (150–152). In contrast, IL-33 can also support tissue homeostasis and repair mediated by Tregs, which express ST2 predominantly in nonlymphoid tissue (153, 154).

### IL-33 in Skin

Epidermal keratinocytes are the predominant producer of IL-33 while also expressing ST2 on their surface (132, 155). Dermal fibroblasts and macrophages can also produce IL-33 (104). IL-33 has been reported to be highly expressed in several skin diseases, including atopic dermatitis (155), psoriasis (156), and vitiligo (157). Serum IL-33 levels are higher in atopic dermatitis patients compared with healthy individuals and it correlates with excoriation and xerosis scores in atopic dermatitis (158). On the other hand, an increase in IL-33 is observed in skin lesions of psoriasis while no increase is observed in the serum (158). ST2 is distributed widely on dermal immune cells, including Th2 cells, Tregs, ILC2s, and mast cells. IL-33 is released from keratinocytes exposed to the invading pathogens, such as *Staphylococcus aureus* and house dust mite allergens, to instigate cutaneous immunity (159, 160). Environmental insults, such as ultraviolet B radiation and hypo-osmotic stress, and mechanical injury, also trigger the induction of IL-33 in keratinocytes (161–163). Interferon (IFN)-γ and tumor necrosis factor (TNF)-α are other known inducers of IL-33 in keratinocytes (164, 165). Nuclear IL-33 is elevated in human keratinocytes stimulated by TSLP and is required for TSLP-mediated suppression of epidermal barrier integrity components, indicating that nuclear IL-33 is a key mediator for chronic TSLP-induced skin barrier dysfunction (166).

IL-33 is implicated in type 2 immune response and the pathogenesis of allergic inflammatory diseases, such as atopic dermatitis (167). Excess IL-33 release from keratinocytes activates ILC2s to produce IL-5 and IL-13, which induce the accumulation of eosinophils in the dermis (168). Basophils induced by IL-33 also boost the ILC2 function *via* IL-4 signaling (169). IL-33 increases histamine generation in mast cells through p38 activation (170). Furthermore, IL-33 is involved in the induction of systemic allergic inflammation as keratinocyte-derived IL-33 can mediate skin-gut crosstalk culminating in the expansion of intestinal mast cells through ILC2 activation and food anaphylaxis (163, 171).

IL-33/ST2 signaling activates sensory neurons to mediate itch and pain responses (172–174). Excessive release of IL-33 from keratinocytes irradiated with poison ivy-derived allergen urushiol enhances the calcium influx in dermal peripheral dorsal root ganglia neurons through its receptor, ST2, to evoke itch and inflammatory responses (172). Neuronal ST2 signaling is a critical regulator of the development of the dry skin itch, but not an itch associated with atopic dermatitis (173). On the other hand, pathogen-derived lipopeptides, such as fibroblast-stimulating lipopeptide-1, can activate TLR2 in dorsal root ganglia, which, in turn, leads to infiltration of macrophages and release of IL-33 from keratinocytes. IL-33 activates the nociceptive sensory neurons at the superficial layers of the skin to instigate and prime the inflammatory pain responses (174). IL-33-mediated molecular mechanisms responsible for itch and pain are an active area of investigation.

IL-33 also plays a regulatory role in the inflamed tissue to restrain inflammation and promote remodeling in the skin, at least in part through the regulation of Tregs and M2 macrophages (175, 176). IL-33/ST2 signaling induces the expansion of Tregs, which have potent anti-inflammatory activity (177). IL-33 release from keratinocytes following skin barrier disruption induces antigen-specific Tregs to suppress excessive skin inflammation in a model of contact hypersensitivity (175). The diabetic mice model shows that IL-33 enhances extracellular matrix deposition and angiogenesis through the polarization of M2 macrophages to promote wound healing (176). Dysregulation of IL-33-mediated Treg induction causes aberrant chronic inflammation and fibrosis in the skin (151, 178). Furthermore, skin-resident Tregs from patients with systemic sclerosis are differentiated into Th2-like Tregs, which produce a higher amount of IL-4 and IL-13, by high expression of skin-localized IL-33, suggesting that IL-33 might be an important factor that contributes to fibrosis due to loss of normal skin-localized Tregs function (98). Single-cell RNA sequencing analysis of skin murine Tregs reveals a predominance of Th2-like Tregs, which preferentially express high levels of the master Th2 transcription factor, GATA3, and are more differentiated into cells, which have tissue reparative capacity (179). GATA3^+^ Tregs in skin express ST2, which enables them to enact reparative functions in response to alarmin IL-33 (180). Thus, IL-33/ST2 signaling has diverse impacts on skin-resident Tregs and their function in the steady-state and fibrosis development.

## Beyond the Role of Type 2 Cytokines as Alarmins in Skin Health

**Figure 1:**
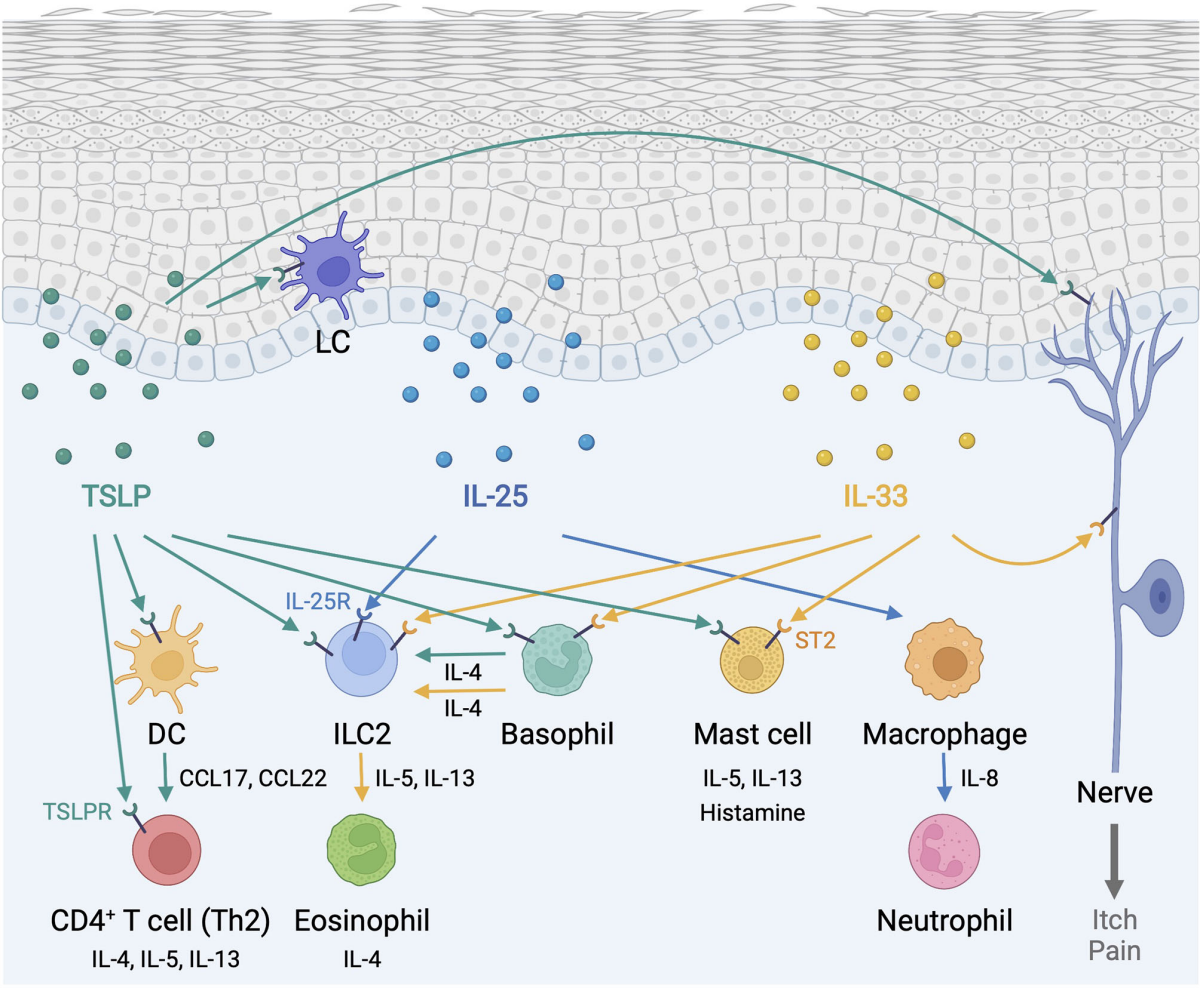
Type 2 alarmin cytokines orchestrate immune responses in the skin. Epidermis-derived TSLP, IL-25, and IL-33 act as alarmins to instigate cutaneous type 2 immunity through a complex and pleiotropic network of innate and adaptive immune cells. In addition, TSLP and IL-33 provoke sensory neurons to mediate itch and pain responses. DC, dendritic cell; ILC2, group2 innate lymphoid cell; LC, Langerhans cell; TSLP, thymic stromal lymphopoietin; TSLPR, thymic stromal lymphopoietin receptor. Created with BioRender.com.

**Figure 2:**
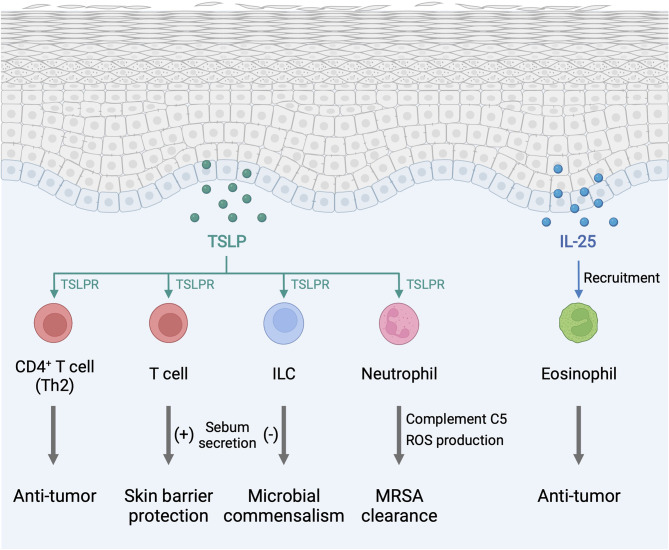
Type 2 alarmin cytokines exhibit pro-homeostatic properties in the skin. The function of type 2 alarmin cytokines extends beyond their conventional Th2-associated function in host defense and allergy. In the skin, these cytokines play critical roles in cancer regulation and sebum secretion. ILC, innate lymphoid cell; MRSA, methicillin-resistant *Staphylococcus aureus*; ROS, reactive oxygen species; TSLP, thymic stromal lymphopoietin; TSLPR, thymic stromal lymphopoietin receptor. Created with BioRender.com.

TSLP, IL-25, and IL-33 alert the immune system in the skin to respond to environmental insults, and their chronic overexpression triggers allergic inflammation at barrier sites (**Figure 1**). Importantly, the function of type 2 alarmin cytokines extends beyond their physiological function in host defense and pathological function in allergic inflammation and involves other critical roles including skin cancer regulation and sebum secretion (**Figure 2**). Keratinocyte-derived TSLP protects the skin from carcinogenesis (181, 182). TSLP exerts its dominant anti-tumor effects through the induction of CD4^+^ Th2 cell immunity in the early stages of keratinocyte cancer development (181). In contrast, TSLP induces proliferation of the malignant CD4^+^ T cells in CTCL lesions, which are marked by a Th2 cell-dominant phenotype in advanced stages (183). It has also been shown that TSLP can recruit IgE-bearing basophils into inflamed skin, and IgE promotes inflammation-driven tumor growth during chronic tissue inflammation in a cutaneous squamous cell carcinoma model (184). Baseline TSLP expression in breast and pancreatic cancer has been linked to a pro-tumorigenic function (185–188). A tumor-myeloid cell axis independent of T cell response may mediate this tumor-promoting function of TSLP (189). However, systemic TSLP induction from the skin causes an effective CD4^+^ T cell-mediated anti-tumor immune response at the site of developing cancer in the breast (185). Topical treatment of calcipotriol, a TSLP inducer (75), blocks skin cancer development in mice in a TSLP-dependent manner, and synergistically with 5-fluorouracil (5-FU) induces an effective CD4^+^ T cell-mediated immunity against actinic keratosis, which is a precursor to cutaneous squamous cell carcinoma in humans (190). IL-25 also has potent anti-tumor effects against several tumor types, including melanoma, through an increase in eosinophils recruitment into the tumor (191, 192). In other models, IL-25 itself exhibits anti-tumor activity through the induction of apoptosis in cancer cells without affecting nonmalignant cells (193). In contrast, epidermal IL-33 contributes to a microenvironment that supports tumor growth and progression in murine skin. Nuclear IL-33 mediates focal adhesion kinase (FAK)-dependent secretion of soluble ST2, a decoy receptor, and CCL5 from squamous cell carcinoma cells, which stimulates immunosuppressive Tregs leading to cancer immune evasion (194). Continuous IL-33-driven stimulation of Tregs shapes a tumor-promoting immune environment associated with chronic inflammation in the murine skin, and an increase in IL-33 expression and Treg accumulation are observed in the perilesional skin of patients with cancer-prone chronic inflammation (178). Furthermore, IL-33 induces the recruitment of a subset of tumor-associated macrophages, which express ST2 and high-affinity IgE receptor, FcϵRIα, and produce TGF-β, in the tumor microenvironment, which results in tumor progression in a mouse model of squamous cell carcinoma (195). Nuclear IL-33 in keratinocytes also promotes intrinsic TGF-β signaling through the SMAD signaling pathway, which constitutes a cell-autonomous tumor promotion mechanism in chronic inflammation (196). IL-33-stimulated macrophages highly produce MMP-9, which proteolytically trims activating receptor natural killer group 2, member D (NKG2D) and its ligands MHC class I polypeptide-related sequence A/B (MICA/B) on the surface of tumor-infiltrating lymphocytes and melanoma cells, and thus impedes the immune surveillance of tumor-infiltrating lymphocytes (197). These findings indicate that type 2 alarmin cytokines have distinct mechanisms for the regulation of cutaneous malignancies.

Endogenous TSLP controls the steady-state level of sebum secretion and sebum-associated antimicrobial peptide expression through the activation of T cells in murine skin. TSLP overexpression results in loss of white adipose tissue in conjunction with sebum hypersecretion (18). TSLP helps to maintain skin-resident RORγt^+^ ILCs within hair follicles, and, by the virtue of their location, ILCs negatively regulate surrounding sebaceous gland size and lipid content to regulate commensal bacteria equilibrium and fine-tune the skin barrier surface in mice (198). These findings suggest that TSLP-elicited ILCs and T cells play opposing functions in sebum secretion. Finally, TSLP is found to activate neutrophils to protect the skin from infection by methicillin-resistant *Staphylococcus aureus* (MRSA) (199).

## Conclusion

Epidermis-derived TSLP, IL-25, and IL-33 act as alarmins to instigate cutaneous type 2 immunity through a complex and pleiotropic network of innate and adaptive immune cells. Accumulating evidence indicates that these type 2 alarmin cytokines not only function in concert but also have distinct physiological functions. Thus, the panoramic view of the communications between keratinocytes and immune cells through type 2 alarmin cytokines is required to fully understand how these cytokines regulate cutaneous immunity. The function of type 2 alarmin cytokines partly depends on the nature of the inflammatory stimulus, the presence of supporting cytokines and chemokines, and the surrounding microenvironment. Recent studies indicate that type 2 alarmin cytokines exhibit not only conventional type 2 inflammatory properties but also pro-homeostatic properties in the skin. Therefore, further understanding of the spectrum of biologic processes regulated by type 2 alarmin cytokines will provide new insights for the development of effective therapeutic approaches to combat allergic inflammation while utilizing the beneficial effects of these cytokines in the skin.

## Author Contributions

All authors contributed to the writing of the manuscript and approved the submitted version.

## Conflict of Interest

TH is an employee of Shiseido Co. Ltd.

The remaining author declares that the research was conducted in the absence of any commercial or financial relationships that could be construed as a potential conflict of interest.

## Publisher’s Note

All claims expressed in this article are solely those of the authors and do not necessarily represent those of their affiliated organizations, or those of the publisher, the editors and the reviewers. Any product that may be evaluated in this article, or claim that may be made by its manufacturer, is not guaranteed or endorsed by the publisher.
